# Social interaction, occupational engagement, and related factors among older mental health service users with severe mental illness – an explorative cross-sectional study

**DOI:** 10.1186/s40359-026-04142-z

**Published:** 2026-02-10

**Authors:** Carina Tordai, Steven Schmidt, Elisabeth Argentzell, Mona Eklund

**Affiliations:** Department of Health Sciences, Lund University, Box 157, Lund, SE-22100 Sweden

**Keywords:** Aging, Occupational engagement, Severe mental illness, Social interaction

## Abstract

**Background:**

Although research shows that social interaction is related to occupational engagement among adults with mental illness of all ages, this relationship is underexplored among older mental health service users. Factors suggested as relevant for such relationships are, for example, social network characteristics, anxiety/depression, physical problems, and gender.

**Aim:**

This study aimed to explore how people aging with a mental illness, diagnosed at a younger age, perceived social interaction and its relationship with occupational engagement. Additionally, it addresses the influence of perceived symptoms of anxiety/depression, perceived physical problems, and gender.

**Materials/methods:**

An explorative quantitative cross-sectional study was performed with forty-nine mental health service users at retirement age. Self-report questionnaires regarding occupational engagement, social interaction, perceived symptoms of anxiety/depression, and a background questionnaire including questions on physical problems were used. Data was analyzed using conventional statistical analyses, including Pearson correlations and linear regression analysis.

**Results:**

Most participants reported having a friend. However, the results showed that participants’ networks were primarily relying on family for emotional support. Social interaction, anxiety/depression, physical problems, and gender accounted for 21% of the variation in occupational engagement, with anxiety/depression being the only significant variable. No significant gender differences in social interaction or network size were found. For women, perceived anxiety/depression and social interaction were significantly correlated with occupational engagement.

**Conclusions/significance:**

Despite regular access to a social network through activity-based mental health services, the participants had small social networks that lacked diversity. Perceived symptoms of anxiety and depression were negatively related to occupational engagement, and social and emotional factors appeared to impact women’s occupational engagement more than men’s.

## Introduction

People living with severe mental illness often experience difficulties in engaging in meaningful occupations in daily life and also report limited opportunities for social interaction. Previous research has demonstrated a close relationship between social interaction and everyday occupations among mental health service users of all ages [1–5]. Occupations here are understood as the range of activities people engage in during the day, including work, household chores, pastimes, and caring for oneself and others. It has been argued that social interaction is integral to what is termed occupational engagement, that is, the types and quantity of occupations, how occupations are structured in time and place, and the various personal, environmental, and occupational contexts in which they are performed [6]. Although there is a certain body of research supporting that social interaction plays a role in occupational engagement among people with severe mental illness of all ages [2, 7], no research has focused on this topic among mental health service users who are reaching retirement age.

A person’s social interaction is facilitated by their social network, which includes all the individuals the person interacts with, such as household members, friends, and neighbors [8]. According to Palumbo et al. (2015), individuals with severe mental illness often have social networks dominated by family and relatives [9]. Research involving people with severe mental illness of middle age and younger (< 50) found that higher age was generally related to larger network sizes [4]. By contrast, another study reported that the social networks of service users aged 55 years and older tended to diminish [10], also leading to reduced possibilities for social interaction. More recent research has reported on loneliness, small networks [11–13], and challenges associated with relationships [14] among older service users. Still, the size and quality of social networks of people living with severe mental illness aging into retirement age remain to be investigated. According to Smart et al. (2021), social networks are considered essential for aging well with a diagnosis such as psychosis, since having a network with social support also encourages engagement in occupations [15]. Having a social network has also been reported as important for maintaining a sense of identity among older adults diagnosed with mental illness at a younger age [16]. This indicates that maintaining one’s social network is crucial for many aspects of well-being for this group of older adults. Maintaining a social network may be facilitated by engaging in satisfying everyday occupations, as suggested in studies that point to a mutual relationship between social networks and subjective perceptions of occupations [1, 7, 17]. Fingerman and colleagues found that among older adults in general, those with more diverse social interactions had greater occupational engagement than those with fewer social interactions, and interactions within one’s peripheral social network were crucial in promoting increased activity levels [18].

An aspect that may be relevant in the relationship between social interaction and occupational engagement is gender. While research on older individuals with mental illness is limited, a study on mental health service users in general reported that women have larger social networks than men [19]. Moreover, research on the general population shows that men and women may place different levels of importance on various aspects of social networks and interactions. For instance, while a strong network of friendships enhanced the psychological well-being of middle-aged adults, kinship networks were more crucial for men’s well-being than for women’s [20]. Similarly, men were more likely to consider their partner as their closest confidant and be more selective in their social relationships than women, who sought support from a broader range of sources [21]. Shin and Park (2023) found that women were more affected by conflict or a lack of support in relationships compared to men [22].

In addition to gender, common psychiatric symptoms should be considered when investigating the social networks of service users. Individuals with mental illness frequently experience symptoms of anxiety and depression, which are prevalent across most diagnoses [23, 24] and often occur in combination. Further, the risk of developing symptoms of anxiety and depression increases with age due to social, physical, and cognitive changes [25], and the occurrence of these symptoms can potentially affect how a person perceives and reports their social interactions and occupational engagement. It is also well established that aging with severe mental illness often results in a range of physical challenges [14, 26–29], not only due to aging but also because of the long-term use of medications with side effects [14]. Perceived physical problems thus constitute another factor to consider in relation to occupational engagement.

Taken together, existing research regarding the connection between social interaction and occupational engagement in older people with severe mental illness is lacking. Filling this knowledge gap is crucial for developing relevant support for this group. As indicated above, gender, degree of anxiety and depression symptoms, and physical problems should be accounted for in research addressing social interaction and occupational engagement. This study aims to explore the relationships between occupational engagement, social interaction, perceived symptoms of anxiety/depression, and perceived physical problems among men and women aging with a severe mental illness, proceeding from the following research questions:

- How do people aging with severe mental illness perceive social interaction?
- How are their perceptions of social interaction, perceived symptoms of anxiety/depression, and perceived physical problems related to their occupational engagement, and how do those relationships vary by gender?

## Materials and methods

This explorative quantitative cross-sectional study was performed in a naturalistic care environment within a larger mixed-methods project focusing on people aging with severe mental illness, from an activity perspective. Explorative research is recommended when there is insufficient knowledge within the field to state well-founded hypotheses [30]. A regional ethical review board approved the study (reg. no. 2013/456), which followed Swedish legislation [31] and the Declaration of Helsinki [32].

### Study setting and recruitment

Data was collected between June 2017 and February 2024. The prolonged timeframe was due to the COVID-19 pandemic, during which data collection was temporarily set on hold. We sought to recruit people aged 60 years and older, based on the year of birth, in order to include participants both before and after retirement age. Eligible participants had a mental illness diagnosis and were attending an activity-based mental health service. Exclusion criteria for prospective participants included having a diagnosis of dementia, having an intellectual disability, or not being able to communicate in Swedish.

A consecutive sampling strategy was employed, meaning that all individuals who met the inclusion criteria during the recruitment period were invited to participate. They were recruited from two types of activity-based services situated in a region of southern Sweden, representing both urban and rural areas. One type of service was municipal-run day centers for people with mental illness. All 33 municipalities in the region were contacted to determine if they had attendees in the stipulated age span in their day center services. Thirteen municipalities responded positively, and since one municipality had more than one day center, a total of 14 day centers were identified. The other type of service approached was occupational therapy provided in general psychiatric units. Two clinics were strategically selected: one mental health open-ward clinic and one open-ward clinic specializing in psychosis, both of which admitted older patients. Within the psychiatric open-ward clinics, participants were identified among those who had either recently received or were currently receiving occupational therapy.

Local staff members at the community-based day centers and at the psychiatric settings were assigned to manage the recruitment process. They received information about the study and invited clients who met the specified inclusion and exclusion criteria. The principle of informed consent was implemented, ensuring that prospective participants received both oral and written information. This information highlighted the principles of voluntariness, the confidentiality of data, and the participant’s right to withdraw at any time. Participants who completed the data collection received a gift card of 100 SEK. The 14 day centers had only a few attendees aged 60 years and above, and a total of 39 individuals from that type of service participated. Sixteen patients from the open-ward clinics were invited, of whom 10 chose to participate.

### Participants

The mean age of the 49 (29 women, 20 men) participants was 64 years. A majority (71%) of the participants were single, and having children was more common than not. The most common educational background was high school completion, and a diagnosis of anxiety or depression was the most frequent diagnostic grouping. A majority reported having physical problems, mainly in terms of pain and limited mobility. Further characteristics are presented in Table 1. No statistically significant differences were found between women and men.

**Table 1:** Socio-demographic and clinical characteristics of the 49 participants

Characteristics	Participants	Gender; *n* (%)	
		Female 29 (59)	Male 20 (41)
Age; mean (min-max)	64 (59–76)	65 (59–76)	64 (60–69)
Civil status; n (%)			
Married/cohabiting	14 (29)	11 (38)	3 (15)
Single	35 (71)	18 (62)	17 (85)
Education level; n (%)			
Completed 9 years of compulsory school	16 (33)	10 (35)	6 (30)
Completed gymnasium	20 (41)	12 (41)	8 (40)
University or college degree	13 (26)	7 (24)	6 (30)
Having children; n (%)	36 (74)	23 (79)	13 (65)
Psychiatric diagnosis; n (%)			
Psychosis	17 (35)	10 (35)	7 (35)
Anxiety/Depression	28 (57)	16 (55)	12 (60)
Other *	4 (8)	3 (10)	1 (5)
Self-reported physical problems; n (%)			
Yes	35 (71)	21 (72)	14 (70)
No	14 (29)	8 (28)	6 (30)
Anxiety/Depression symptom scores; mean (min-max)	13.8 (0–31)	13.6 (0–31)	14 (0–26)

*Other includes, e.g., ADHD, EUPD (emotionally unstable personality disorder), and Mental and behavioral disorders due to multiple drug use

### Data collection

The data was collected by the first author, who is an occupational therapist with vast experience working with older people with severe mental illness. The research team had received permission for all instruments used, which addressed social interaction, subjective experiences of occupational engagement, self-reports of anxiety and depression, as well as socio-demographic and clinical factors. The instruments were administered by the data collector through a dialogue with the participant, during which the data collector read the questions and available answer options. Each data collection lasted approximately 60 min. Breaks were provided as necessary. The data collection was conducted in a booked room either at the day center or the open ward where the participant attended. In a few cases, the data collection took place in the participant’s home. Some participants required assistance in understanding certain scale items. In such cases, the data collector was careful not to influence how the participant responded.

#### Instruments 

##### Background questionnaire

A background questionnaire was used for the socio-demographic factors. It was designed for the study and covered aspects such as gender, age, educational level, civil status, and whether the respondent perceived any physical problems. Additional questions inquired about self-reported diagnosis, perceived psychiatric problems, and psychotropic medication. The diagnoses were subsequently coded by a psychiatrist, following the ICD-10 classification [33]. To capture facets of social interaction, additional questions included whether the participant had a friend and if they had met with a friend in the preceding week.

##### Interview schedule for social interaction – self rating

Social interaction was further assessed using the Swedish version of the Interview Schedule for Social Interaction (ISSI) [34]. The original ISSI has four subscales, two of which concern the availability of social interaction. One targets the availability of social integration (AVSI, six items), and the other targets the availability of attachment (AVAT, six items). The AVSI subscale was deemed particularly valid and reliable for individuals with severe mental illness [35]. It was designed to measure the quantity of a person’s social contacts [34] and asks for the numbers of certain types of contacts, for example, how many people the participant knows who share their interests. The response format is to tick one of six given intervals: none, 1–2, 3–5, 6–10, 11–15, 16 and above. The first three categories are recorded as zero, while the last three are recorded as one [34]. The availability of attachment (AVAT) items inquire if the participant has access to someone close, for example, if they have a confidant with whom they can share their deepest feelings and trust completely. The response alternatives are yes (= 1) or no (= 0).

A brief version, here termed ISSI-bref, which combines five of the integration items (AVSI) with two of the attachment items (AVAT), was used in the current study to measure the availability of social interaction. It renders a possible score between 0 and 7 and has been found relevant for use with people with mental illness [19]. The internal consistency reliability based on the current sample was alpha = 0.68, thus slightly below the stipulated minimum value of 0.70 [36].

In addition to the numerical ratings, each of the two AVAT items in the ISSI bref is followed by an open-ended question asking which persons provide the type of attachment described (having someone with whom they can share their happiness and having a confidant with whom they can share their deepest feelings).

One of the AVSI items, addressing how many acquaintances the respondent meets or speaks to every week, was analyzed separately to indicate social network size. In this case, the initial six intervals were maintained and recoded into an ordinal scale from 0 to 5.

##### Profiles of occupational engagement in people with severe mental illness (POES-S)

Profiles of Occupational Engagement in People with Severe Mental Illness (POES) is an instrument measuring time use and occupational engagement [37]. The first part involves the completion of a 24-hour time-use diary sheet for yesterday, and the second part is a questionnaire with nine items reflecting activity engagement, such as the daily rhythm of activity and rest, and the variety of activities. There is support for POES’s content validity and internal consistency, and it is a good fit to the Rasch model [37, 38]. A self-report version, POES-S, was used for the current study. It includes nine statements that examine the following aspects of occupational engagement: the daily rhythm of activity and rest, variety and range of occupations, place, social environment, social interplay, interpretation, meaningful occupations, routines, and initiating performance. Each statement has the following alternatives to choose from: does not apply at all (= 1), hardly applies (= 2), partially applies (= 3), and fully applies (= 4). The total score on POES-S ranges from 9 to 36, with a higher score indicating greater activity engagement. No psychometric study has been published on POES-S, but Cronbach’s alpha based on the current data was α = 0.80, and all item-total correlations were > 0.30.

##### Hospital anxiety and depression scale (HADS)

The Hospital Anxiety and Depression Scale (HADS) is a self-report of anxiety and depression symptoms, and it gives a measure of the patient’s mood in the last week. It includes 14 statements, such as: *Worrying thoughts go through my mind* from the anxiety subscale, and *I still appreciate the things I used to appreciate*, from the depression subscale, which the respondents are expected to respond to. Both the anxiety subscale and the depression subscale contain seven intermingled items each, and the items are rated on a 4-point scale ranging from 0 (not present) to 3 (considerable). The instrument has good screening properties for the identification of anxiety disorders and depression [39]. HADS is also found to perform well in assessing the symptom severity of anxiety disorders and depression in both somatic, psychiatric, and primary care patients and the general population [39]. To capture the current level of symptom severity, the summed score of anxiety and depression symptoms was used in the current study.

### Data analyses

Descriptive statistics were used to delineate the participants’ situation concerning social interaction. Parametric statistics were used for composite scales, which were considered to be approximately at the interval scale level. Pearson correlations, two-tailed, were employed to calculate correlations between variables, stratified by gender when appropriate. In line with Akoglu [40], the limits for the strength of correlations were set as “very strong” for *r* ≥ 0.90, “strong” for 0.70 > *r* < 0.89, “moderate” for 0.40 > *r* < 0.69, “weak” for 0.10 > *r* < 0.39, and “negligible” for *r* < 0.10. Multiple linear regression analysis was used to analyze which independent variables were important to occupational engagement. The independent variables were checked for multicollinearity, and correlations ranged from − 0.30 to 0.20, indicating that multicollinearity was of no concern. Normal distribution of residuals was ensured through graphical examination. The Chi-square test was used to analyze categorical variables. An independent samples t-test was conducted to compare the availability of social interaction between genders. Concerning the one-item ordinal scale based on the single AVSI item indicating social network size, non-parametric statistics were seen as appropriate. Spearman correlations were used to calculate associations between variables, and the Mann-Whitney test was used to evaluate gender differences on this variable. Responses to the two open-ended AVAT items were categorized based on similarities in three categories: family, friends, and staff/other. The categorization was made by the first and last author separately, and the agreement was complete. The software used was SPSS 29 [41].

## Results

### Availability of social interaction

Thirty-six (73%) participants indicated they had a friend, and 30 (61%) stated they had met a friend in the last week. When participants were asked if they had someone in particular with whom they could share their feelings of happiness, 30 (61%) participants answered yes, with one participant not specifying who. The individuals they confided in could be categorized into three groups: family (20), friends (7), and staff/other (2). In response to the question about whether they had someone in particular with whom they could share their innermost feelings, 29 (59%) participants responded affirmatively. Their answers fell into four categories: family (16), friends/neighbors (7), and staff/other (6).

Among the 49 participants, 41 (84%) reported meeting or speaking with ten people or fewer each week, and 22 (45%) reported interacting with five people or fewer.

### Variables related to occupational engagement

Participants’ total scores on occupational engagement (POES-S) ranged from 17 to 36, with a mean score (SD) of 28.6 (4.9). Availability of social interaction (ISSI-bref), self-report of anxiety/depression symptoms, gender, and physical problems (having/not having) were entered into a linear regression analysis with occupational engagement (POES-S) as the dependent variable. The results indicated that the model explained 21% of the variation in occupational engagement (adjusted R square = 0.21, p for F-test = 0.006). As indicated in Table 2, anxiety/depression was the only single independent variable that was statistically significant concerning occupational engagement.

**Table 2:** Results from the linear regression analysis with occupational engagement as the dependent variable

Independent Variable	Unstandardized beta value	95% confidence interval for B	*P*-value
		Lower Upper	
Anxiety/Depression	−0.290	−0.465 −0.115	0.002
Gender	−1.334	−4.023 1.355	0.323
Social interaction	0.423	−0.414 1.261	0.313
Physical problems	−0.830	−3.707 2.047	0.564

The correlation between the single AVSI item addressing social network size and occupational engagement was on the border of statistical significance, r_s_=0.27 (*p* = 0.05).

### The role of gender

The distribution of women’s and men’s ratings on the availability of social interaction (ISSI-bref) is displayed in Table 3. Regarding the eight possible sum scores (0–7), 75% of men and 55% of women scored on the lower end (0–3). No statistically significant gender difference was found, t=−1.54 (*p* = 0.131).

**Table 3:** The distribution of women’s and men’s scores on the social interaction scale

Scoring	Women		Men	
	Frequency (%)	Cumulative percent	Frequency (%)	Cumulative percent
0	1(3)	3	2 (10)	10
1	5 (17)	21	4 (20)	30
2	10 (35)	55	9 (45)	75
3	6 (21)	76	4 (20)	95
4	2 (7)	83		
5	2 (7)	90		
6	1 (3)	93		
7	2 (7)	100	1 (5)	100

Additionally, there was no gender difference on the item addressing social network size (z=−0.67, *p* = 0.501). When comparing men and women regarding which category of supporting contacts they had, no gender differences were found for having someone to share their happiness with (chi^2^=1.92, *p* = 0.382) or having a confidant to share one’s deepest feelings with (chi^2^=1.83, *p* = 0.913).

Correlations between the availability of social interaction (AVSI brief) and occupational engagement (POES) and between anxiety/depression (HADS) and occupational engagement were calculated for men and women separately and are presented in Table 4. No statistically significant correlations were found between the targeted variables among the men. In the subgroup of women, the relationship between symptoms of anxiety/depression and occupational engagement was statistically significant, as was the relationship between the availability of social interaction and occupational engagement.

**Table 4:** Pearson correlations from the availability of social interaction and anxiety/depression to occupational engagement, split by gender

Gender	Variable	Occupational engagement (*r*)	*P*-value
Women	Social interaction	0.44	0.017
	Perceived symptoms of anxiety/depression	−0.68	0.001
Men	Social interaction	0.02	0.933
	Perceived symptoms of anxiety/depression	−0.15	0.542

## Discussion

A majority of the participants reported having a friend, but despite regular access to a social network through activity-based mental health services, they had small networks and relied mainly on their family for personal confiding. The regression model accounted for 21% of the variability in occupational engagement, with perceived symptoms of depression/anxiety as the only statistically significant variable. There were no significant differences between genders in terms of the availability of social interaction or the size of their social networks. However, a difference did arise regarding factors related to occupational engagement. For women, anxiety and depression, along with the availability of social interaction, were associated with their occupational engagement, while these factors did not have the same relationship with occupational engagement among men.

Almost half of the individuals reported having no one to confide in, even though they felt they had friends. Those who had someone to confide in primarily turned to family members for emotional support. Thus, despite having friends and access to the social network available at the activity-based mental health services, many participants relied on family members for emotional support and confiding. This is consistent with previous research on adult mental health service users, both younger than the age of 65 [1, 9] and older [10], and suggests that although activity-based mental health services offer opportunities for social interaction, they do not inherently promote the development of closer interpersonal relationships.

The participants in this study reported limited social networks, despite having access to services that, according to previous research, may facilitate social contacts [1]. Nearly half of the participants reported interacting with no more than five people per week, which aligns with previous research of older people living with severe mental illness [11–13], but contrasts with the recent estimate of an average network size of 15 individuals among older adults from the general population in Sweden [42]. Those without access to activity-based mental health services, a group not reached in the current study, might have even smaller social networks. Also, recent research shows that it is common that service users tend to disengage from activity-based services as early as around the age of 50 [43] and are then without this form of support. This highlights the need for further research addressing the social networks of older mental health service users. It is also important to increase the knowledge about those who choose to leave activity-based services and to find ways to encourage them to stay connected.

Furthermore, given that many participants lived alone, which may be typical for those aging with severe mental illness, it seems crucial to prioritize expanding social networks and close social relationships in care planning. Integrating peer support workers, i.e., individuals with lived mental health experience, can foster these relationships and aid recovery [44]. While peer support workers are part of Swedish mental health care [45], expanding their role to support older adults with severe mental illness is recommended.

Occupational engagement is a complex phenomenon that may be influenced by multiple factors. The regression model performed in the current study showed that perceived symptoms of anxiety/depression were related to occupational engagement, aligning with previous research on people with severe mental illness [46]. However, additional factors, not included in the model, might also influence occupational engagement among the participants, warranting further research to address this issue. Self-mastery and time spent at the activity-based mental health services, which were not included in our model, have previously been reported as important factors influencing occupational engagement [46]. Furthermore, the influence of factors such as gender and physical problems might deserve more detailed exploration.

Diagnoses of anxiety or depression accounted for more than half of all diagnoses among the participants. Moreover, anxiety and depression are often prevalent additional diagnoses among individuals with psychosis [23, 24], which was the second most common diagnosis in this sample. Social isolation and aging are known to increase the risk of depression [47], making it crucial to focus on interventions that prevent and reduce anxiety and depression in older adults with severe mental illness. Accordingly, the World Health Organization [47] highlights the importance of maintaining social connections and engaging in meaningful activities in later life. Similarly, research has emphasized that having larger, more diverse social networks and closer relationships can help alleviate symptoms of depression [48]. Additionally, research has elucidated that individuals without depressive symptoms tend to have a higher level of social interaction [1, 4]. Thus, engaging in meaningful activities and social connections may be crucial to prevent and reduce anxiety and depression among older adults, and specific interventions for that purpose are urgently needed. For example, it would be possible to further develop and adapt interventions such as the Balancing Everyday Life program [49] and the Well Elderly program [50] to better meet the needs of older individuals with mental illness.

The relationship between social network size (the number of people the participants interacted with last week) and occupational engagement was on the verge of statistical significance in the present study. This tendency (*p* < 0.1), although not statistically significant, suggests that facilitating access to social connections in mental health care services would be important, particularly since relationships between social networks and occupational engagement have been shown in prior research. Specifically, a larger and more diverse social network was associated with greater occupational engagement among older adults in general [18]. The fact that the participants in the current study had small and not-so-diverse networks may be a reason why there were no significant relationships between social network size and occupational engagement or between social interaction and occupational engagement.

Both men and women reported low levels of availability of social interaction, and there was no statistically significant difference between women and men, which is inconsistent with previous research reporting that female mental health service users tend to have larger social networks than their male counterparts [19]. In the current study, there was a tendency for men to live without a partner to a greater extent than women (*p* < 0.1). Related to this, previous research has shown that living with someone entails more activities in the older population [51]. Given that social interaction in the present study was related to occupational engagement only among the women and not among the mostly single men may be influenced by the same dynamics. A recent systematic review [52] and previous research [20, 22] have shown gender differences in various aspects of social networks and interaction. Furthermore, anxiety/depression was related to occupational engagement only among the women, which suggests gender differences regarding the importance of emotional factors for occupational engagement. Hence, there is a need for deeper insights into the roles of social and emotional experiences of older men in relation to occupational engagement, which could be achieved through qualitative studies.

### Strengths and limitations

This study was exploratory, given the limited research on the topic. A notable limitation was the relatively small sample size. We aimed to recruit 60 participants, as indicated by a power analysis informed by previous research using the same instruments with individuals diagnosed with severe mental illness [53]. However, although there was an extensive recruiting process, only 49 participants were enrolled through consecutive sampling. This did not reach the target sample size in full and thus increases the risk of type-2 errors. The sample size also constrains the number of variables that can be included in a linear regression model, with a general recommendation of having at least ten participants per independent variable [54]. For this study, the number of independent variables should not exceed four. Variables not included in the current study, such as financial situation, self-mastery, or time spent at the activity-based mental health service, might be of importance and should be given attention in further research on social interaction and occupational engagement. It is also possible that the question about having/not having physical problems used in the current study was too imprecise to reflect the role of physical problems in relation to occupational engagement. The sample size also affected the validity of the statistical results; hence, larger studies are warranted to confirm the results of the current study. Furthermore, the small sample size likely limited the statistical power to find differences between men and women. The fact that data collection was put on hold during the COVID-19 pandemic adds to the robustness of the data. Had it not been interrupted, the widespread increase in loneliness and decrease in activity levels typically seen during the pandemic might have influenced participants’ responses. Moreover, the delay also meant that new individuals could be included at the day centers, thereby expanding the recruitment base. However, despite this advantage, selection bias may have occurred since local staff members identified participants, and those who agreed to participate could differ from those not approached, potentially limiting representativeness.

Something that strengthened the internal validity and dependability of the results was the fact that standardized questionnaires were employed and administered through dialogues with the same data collector for all participants. However, the sample was limited to those who received activity-based mental health services. This limits the external validity, as the findings may not extend to older adults with severe mental illness who are not receiving services and whose circumstances and needs differ. Nevertheless, the findings may serve as a foundation for generating hypotheses for future research.

As this is a cross-sectional study, the results cannot be used to infer a cause-and-effect relationship between social interaction and occupational engagement. It is probable that anxiety and depression, along with occupational engagement, mutually influence each other, which also applies to the relationship between anxiety, depression, and social interaction.

## Conclusion and clinical implications

This study provided new knowledge about social interaction, occupational engagement, and related factors among older people with severe mental illness. Participants had small social networks and relied primarily on family for close relationships, even though they had opportunities for additional social interaction through activity-based services. Many participants lived alone, which is often the case for individuals aging with mental illness, and nearly half of the participants reported having contact with fewer than five people each week, which suggests possible social isolation. Given that social isolation is a significant risk factor for anxiety and depression, it is crucial to focus on supporting opportunities that can help expand the social networks and foster close relationships among older people living with severe mental illness. Additionally, since perceived symptoms of anxiety/depression were found to be related to occupational engagement, and the relationship is likely to be bidirectional, this further strengthens that providing access to activity-based mental health services that include opportunities for social interaction and occupational engagement could be beneficial to enhance well-being as well as prevent and treat anxiety/depression.

Regarding gender, this study did not yield any clear-cut answers but echoes previous research that gender might play a role in which factors are influential concerning social interaction and occupational engagement, and that those may differ between women and men. The findings suggest that social and emotional factors may have a greater impact on occupational engagement for women than for men. Gender is thus an issue that needs to be revisited in future research on social interaction, occupational engagement, and related factors. In particular, future research should investigate which factors are important for social interaction among men.

Future research should involve larger samples to validate the current results and further investigate the connection between social interaction and occupational engagement in older people with severe mental illness.

## Data Availability

The data are available from the corresponding author on reasonable request, but are not publicly available due to restrictions under the Swedish Act concerning the Ethical Review of Research Involving Humans.

## Abbreviations

AVAT: Availability of Attachment
AVSI: Availability of Social Integration
HADS: Hospital Anxiety and Depression Scale
ICD: International Classification of Diseases
ISSI: Interview Schedule for Social Interaction
ISSI-bref: Availability of Social Interaction
POES: Profiles of Occupational Engagement among people with Severe Mental Illness
POES-S: Profiles of Occupational Engagement among people with Severe Mental Illness, Self–report
SDO-OB: Satisfaction with Daily Occupations and Occupational Balance
